# Internet addiction among social networking sites users: Emerging mental health concern among medical undergraduates of Karachi

**DOI:** 10.12669/pjms.346.15809

**Published:** 2018

**Authors:** Zaeema Ahmer, Sana Tanzil

**Affiliations:** 1 *Dr. Zaeema Ahmer, MBBS, MSPH (JSMU). Lecturer, APPNA Institute of Public Health, Jinnah Sindh Medical University Rafiqi H J Road, Karachi, 75510, Pakistan*; 2 *Dr. Sana Tanzil, MBBS, FCPS (AKU). Assistant Professor, APPNA Institute of Public Health, Jinnah Sindh Medical University Rafiqi H J Road, Karachi, 75510, Pakistan*

**Keywords:** Internet Addiction, Medical Undergraduates, Young’s Internet Addiction Test, Social Networking Sites

## Abstract

**Objective:**

To determine the frequency and intensity of Internet Addiction (IA) among medical undergraduates, using Social Networking Sites (SNS), in Karachi.

**Methods:**

A cross-sectional survey was conducted in March-June ‘16 in a private and government medical college of Karachi. Self-administered, Young’s Internet Addiction Test was implemented by 340 medical students to assess the frequency and intensity of IA among SNS profile users for past three years. The structured questionnaire further enquired regarding the social and behaviour patterns relevant to IA and SNS use. Data was analyzed using SPSS 16.

**Results:**

Internet Addiction (IA) was found in 85% (n=289) of all study participants. Among them, 65.6% (n=223) were ‘minimally addicted’, 18.5% (n=63) were ‘moderately addicted’, whereas 0.9% (n=3) were found to be ‘severely addicted’. Burden of IA was relatively higher among female medical students as compared to male medical students (p=0.02). There was no significant difference between type of medical college attended and IA (p=0.45). However, statistically significant differences were observed in certain behavioural patterns among addicted and non-addicted medical students.

**Conclusion:**

Internet Addiction (IA) is an emerging mental health concern affecting social behaviour patterns of medical undergraduates. However, the burden of IA is relatively higher among female medical students.

## INTRODUCTION

Internet has increasingly influenced all aspects of society and the exponential rise of the global users indicates that it has become an integral part of the daily lives of people of modern era.

Ever since its inception, there remains a worldwide controversy on the potentially addictive effects of its overuse.^1^ There has been much universal debate on Internet Addiction (IA), which has resulted in labeling it as a compulsive-impulsive spectrum disorder and meriting its inclusion in the Diagnostic *and Statistical Manual of Mental Disorders, Fifth Edition (DSM-V*).^1^,^2^

As a contemporary venue for building relationships online, the emergence of Social Networking Sites (SNS) has also attracted numerous users across the globe. While such networks allow users, groups and communities with shared interests to stay ‘connected’, the threats it pose in the form of psychosocial addiction is real too. Today the mass appeal of SNS in undergraduate students is a cause of concern as well due to their mass addictive qualities.^3^

Dr. Kimberly Young, a licensed psychologist and an internationally known expert on IA, specified different types of IA as *gaming, web surfing, net compulsions* like online gambling or shopping, pornography and lastly *cyber-relationship addiction* which includes SNS addiction. 4, 5

The universal studies of IA on high school and undergraduate students, between 14 to 24 years, having SNS profiles, found in different countries such as USA, Europe, India, Iran, China, Korea, Sudan etc. reported frequencies ranging between 0.7% to 33%. 6-12 This implies that IA is recognized worldwide and there is a variable burden of IA across the globe.

As far as studies in Pakistan are concerned, limited work has been done to identify medical undergraduate users of SNS, falling victim to IA. A study concluded that in Pakistan, users of networking sites are increasing tremendously.^13^ Among Asia’s top ten internet countries, Pakistan is ranked 7th with 17.5 million internet users and out of them 50% use online SNS.^13^ A cross-sectional study amongst 412 medical students of Agha Khan University, indicated that 74% of the sample was minimal addicts, 24% were moderate addicts and 2% were severe addicts.^14^

### Rationale of the study

Due to the paucity of literature and to highlight the importance of mental health of the youth of a nation; further information is needed regarding the psychosocial impact of excessive SNS and Internet usage. With connectivity becoming so widespread and the ever-evolving SNS, being launched and re-launched, our youth is devoting more time staying online. This is certainly not a disorder but for someone who is vulnerable and has reinforcing environmental variables, it can lead to an increased risk of addictive behaviour. This in turn can pave way for social isolation and deficiency in academics. Henceforth, this study was undertaken to assess the burden and intensity level of IA amongst medical undergraduates, using SNS and this in turn will help us in proposing possible solutions and directing future research towards this newly emerging addiction domain.

## METHODS

A cross-sectional study was conducted between March and June 2016 in a public medical college, SMC, Jinnah Sindh Medical University and a private medical college, Liaquat College of Medicine and Dentistry after taking ethical approval by the Institutional Review Board.

Male and female medical students, who were active SNS users for the past three years were inducted through proportionate quota sampling with an estimating percentage of girls and boys participating in the study as 60% and 40% respectively. Sample size was estimated from the software Open Epi. With an anticipated population proportion of 33.4%, confidence level of 95% and bound on error of 5%, a sample size of 340 was calculated.^15^ SNS included primarily networking sites for entertainment, blogging, picture sharing, employment and future job opportunities and connecting and video-chatting. ‘Young’s Internet Addiction Test’ a validated and self-administered questionnaire was used for this study after getting informed consent. It was developed in 1998 by Dr. Kimberley Young was used for this study after receiving her approved permission through email. It consisted of 20 closed ended items with responses on five points Likert Scale items.^16^ It covered the degree to which internet use affects daily routine, social life, productivity, sleeping pattern, and feelings. The minimum score was 20 while the maximum was 100 and the higher the score the greater the level of IA. Three types of Internet-user groups were identified in accordance with the original scheme of Young. The scores ranging from 20 to 49 indicated minimal addicts, from 50 to 79 indicated moderate addicts and from 80 to 100 indicated severe addicts. Scores from one to 20 reflected a normal level of Internet usage. The instrument exhibited good psychometric properties in previous researches. This test had high face validity and its reliability was 0.899 Cronbach’s Alpha.^17^ Questions regarding the usage pattern of SNS, common reasons of excessive usage and psychosocial behavioural patterns as a result of IA were also addressed.

A student was classified as an internet addict if his score was above 20 and the intensity of minimal, moderate and severe IA was also operationalized on scores according to Young’s criteria.

After pre-testing on 10% of similar sample, data was collected, cleaned for missing variables and cross-validated by random checking. Data was entered in SPSS version 16, where categorical variables were summarized by frequencies and percentages. Chi Square test was used to find the significant difference between gender and public and private institutes with IA. Fisher’s Exact test was used to assess for the significant differences within and between IA groups and the psychosocial behavioural patterns. A p-value of ≤0.05 indicated statistical significance.

## RESULTS

Out of the 340 participants, there were 40% (n=136) males and 60% (n=204) females with equal representation i.e. 50% (n=170) each from both the government and private medical college. Mean age of the participants was reported as 21.20 ±1.67 years.

More than two-thirds i.e. 95% (n =323) of the participants had a profile on Facebook. It was followed by Skype with 60.6% (n=206) users. Main reason for logging on to SNS was to read about latest news update as reported by 69.7% (n=237) of the participants. Stalking was also self-reported by 4.1% (n=14) of them. Frequency of IA was reported as 85% (n=289), with intensities of IA as 65.6% (n=223) minimal addicts, 18.5% (n=63) moderate addicts and 0.9% (n=3) severe addicts. (Table-I)

**Table-I:** Sociodemographic characteristics, patterns of SNS use and frequency of IA of medical undergraduates (n=340).

Variable	n (%)
***Age(years)(Mean: 21.20SD±1.67)***	
19-21	197 (57.9)
22-25	143(42.1)
***Registered with SNS^***	
Facebook	323 (95)
Skype	206 (60.6)
MySpace	12 (3.5)
Instagram	143 (42.1)
LinkedIn	46 (13.5)
Tumblr	27 (7.9)
Twitter	105 (30.8)
Google plus	148 (43.5)
***Reasons of use as reported by students^***	
To play games	68 (20)
To update status	91(26.7)
To check out what’s going on with friends	203 (59.7)
To read latest news update	237(69.7)
To follow their favorite stars	86(25.3)
Others**	14 (4.1)
***Frequency of IA***	
Internet addicts	289 (85)
Not addicted to the internet	51 (15)
***Intensity of IA***	
Minimal addiction	223 (65.6)
Moderate addiction	63 (18.5)
Moderate addiction	3 (0.9)

^ Multiple responses,

* Occasionally include once a month or less

** Others include stalking, studying, reading memes

On comparing frequencies of IA with gender, female participants (57.4%, n=166) were more addicted to the internet as compared to their male counterparts (42.5%, n=123) and this difference was found to be statistically significant (p= 0.02). However, when frequencies were compared across medical college, participants enrolled in government medical college were found to have higher frequencies of IA (43.2%, n=147) as compared to the ones enrolled in private medical college (41.7%, n=142) which was not statistically significant (p=0.45). (Table-II)

**Table-II:** Comparison of frequencies of IA with Gender, Type of Medical College (n=340).

Variable	Internet Addicts n (%)	Not addicted to the internet n (%)	p-value*
Male	123(36.1)	13 (3.8)	0.02
Female	166 (48.8)	38(11.1)	
Government College	147(43.2)	23 (6.7)	0.45
Private College	142(41.7)	28 (8.2)	

* Chi- Square as test of significance, p≤0.05

Differences in certain behavioural patterns were also observed amongst medical undergraduates with and without IA using SNS (Table-III). Between the three groups of non-internet addicts, minimal addicts and moderate to severe addicts, higher frequencies of behavioural patterns were observed in the minimal addicts and the differences were all statistically significant. (p< 0.001)

**Table-III:** Distribution of behavioural patterns among medical undergraduates, who are SNS users, with and without IA (n=336).

Behavioural Patterns due to SNS use	Not addicted to the internet *n* (%)	Minimal addicts *n* (%)	Moderate to severe addicts** *n* (%)	p-value*
***Excessive time expenditure***			
Yes	5(1.4%)	47(13.9%)	24(7.1%)	0.002
No	47(13.9%)	171(50.8%)	42(12.5%)	
***Ignored academics***			
Yes	12(3.5%)	87(25.8%)	47(13.9%)	p<0.001
No	40(11.9%)	132(39.2%)	18(5.3%)	
***Hidden use from family/friends***			
Yes	3(0.8%)	69(20.5%)	33(9.8%)	p<0.001
No	48(14.2%)	151(44.9%)	32(9.5%)	
***Preferred virtual friendship***			
Yes	5(1.4%)	60(17.8%)	34(10.1%)	p<0.001
No	45(13.3%)	159(47.3%)	31(9.2%)	
***Frustration on non-availability***			
Yes	18(5.3%)	131(38.9%)	59(17.5%)	p<0.001
No	33(9.8%)	89(26.4%)	6(1.7%)	
***Source of distraction***			
Yes	31(9.2%)	179(53.2%)	56(16.6%)	p<0.001
No	20(5.9%)	41(12.2%)	9(2.6%)	
***Failed exam and blamed on SNS***			
Yes	6(1.7%)	53(15.7%)	36(10.7%)	p<0.001
No	45(13.3%)	167(49.7%)	29(8.6%)	
***Emotional irregularities***			
Yes	9(2.6%)	84(25%)	44(13.0%)	p<0.001
No	42(12.5%)	136(40.4%)	21(6.2%)	

* Fisher’s Exact test as test of significance, p≤0.05

** Moderate and severe addiction has been combined for statistical purposes.

## DISCUSSION

The findings indicated that 15% were normal users with internet usage within normal limits, whereas a significant number, 85% were found to be suffering from some level of IA. Severe addicts were 0.9%, with significant problems caused by their internet usage for which the role of counseling should be considered to address these problems. Moderate addicts were 18.5%, with frequent problems due to their internet usage and who were at risk of falling in the severe addict’s category. Minimal addicts were 65.6%, who had control over their internet usage but with mild dependence on the internet.

This study finding is consistent with that reported in previous studies done on similar cohort in Nepal and also neighbouring India, which also reported prevalence between 56.5% and 84.6%.^18^-^20^ It must be noted that higher frequencies were reported in somewhat similar study population and with same cultural and social contexts, which could influence the pattern of internet use across these countries. This could be attributed to unlimited and easy access to the internet, introduction of smart phone packages and easy availability of Wi-Fi.^20^ Furthermore, in our part of the world, even though family values are respected but development of nuclear families and emergence of a higher proportion of working parents, leading to lack of parental supervision might contribute to the higher frequencies of IA.^19^ Other factors which need to be explored further are coping with high levels of stress, need for developing intimate relationships, and possibly a lesser family cohesion at home.^20^

The study identified the major reasons for staying and logging on SNS were mainly to read about latest news updates. These results follow the same pattern as the study done in Qassim University, Saudi Arabia where students reported same.^21^ The adoption of SNS as a channel of learning resources by medical students is fundamental to cope with the paradigm shift towards self-directed learning in medical colleges globally and locally. Therefore, it should be encouraged to use SNS, mainly for academic purposes rather than for unnecessary activities. Awareness sessions should be regularly conducted by medical colleges, counseling the medical students regarding excessive use of Internet and SNS.

However, 4.1% students who reported that one of the reasons for logging on SNS was ‘stalking’ warrants attention, as these are warning signs for parents and teachers to forecast the addiction these students will develop over time.

Present study revealed that female medical students were addicted more to the internet as compared to their male counterparts. This corroborates with the study done among Singaporean college students which also reported a significantly higher prevalence in females as compared to males and attributed it to shopping addiction among them.^22^ With the advent of new online shopping websites, IA is becoming more prevalent amongst females and hence this needs to be explored further. However, a possible explanation could be the limited mobility or socialization opportunities among majority of females in our settings due to cultural preferences and preset social norms, affecting their internet usage for the same.

Differences in the three groups of not addicted to Internet, mild to moderately addicted and severely addicted were observed for different behavioural patterns due to SNS use. These included excessive time expenditure, ignoring academics, preference of virtual friendships etc. which were all statistically significant. Even though higher frequencies of behavioural patterns were observed in the minimal addicts as compared to the moderate to severe addicts but this could be due to the small sample size and the sample distribution of the participants included in the study. These results corroborate with study done on undergraduates in a US University which related IA with difficulties in emotion regulation.^23^ Taking responsibilities as young adults is part of the growing up process and one should cope with such emotional stressors without them causing hindrance in a student’s academic performance as well as their social responsibilities towards their families and community.

### Limitations of the study

As a single public and a single private medical college of Karachi was included in this study hence the findings cannot be generalized to entire population of medical students in Karachi. Moreover, it was a self-administered questionnaire implemented by applying non- probability sampling hence the risk of volunteer or interviewer bias cannot be ruled out.

## CONCLUSION

This study observed that there is a high burden of IA in medical undergraduates of selected medical schools in Karachi. The considerable proportion of moderate addicts point towards an emerging burden of internet addiction, and this forecasts a potential rise in severe addicts in the near future. Hence, IA should be considered as an emerging mental health concern.
